# Biological Control of Citrus Postharvest Phytopathogens

**DOI:** 10.3390/toxins11080460

**Published:** 2019-08-06

**Authors:** Jaqueline Moraes Bazioli, João Raul Belinato, Jonas Henrique Costa, Daniel Yuri Akiyama, João Guilherme de Moraes Pontes, Katia Cristina Kupper, Fabio Augusto, João Ernesto de Carvalho, Taícia Pacheco Fill

**Affiliations:** 1 Institute of Chemistry, Universidade Estadual de Campinas, CP 6154, 13083-970 Campinas, SP, Brazil; 2 Faculty of Pharmaceutical Sciences, Universidade Estadual de Campinas, 13083-859 Campinas, SP, Brazil; 3 Instituto Agronômico de Campinas (IAC), 13490-970 Cordeiropolis, SP, Brazil

**Keywords:** biological control, post-harvest phytopathogen, *Penicillium digitatum*, *Penicillium italicum*, *Geothrichum citri-aurantii*

## Abstract

Citrus are vulnerable to the postharvest decay caused by *Penicillium digitatum*, *Penicillium italicum*, and *Geotrichum citri-aurantii*, which are responsible for the green mold, blue mold, and sour rot post-harvest disease, respectively. The widespread economic losses in citriculture caused by these phytopathogens are minimized with the use of synthetic fungicides such as imazalil, thiabendazole, pyrimethanil, and fludioxonil, which are mainly employed as control agents and may have harmful effects on human health and environment. To date, numerous non-chemical postharvest treatments have been investigated for the control of these pathogens. Several studies demonstrated that biological control using microbial antagonists and natural products can be effective in controlling postharvest diseases in citrus, as well as the most used commercial fungicides. Therefore, microbial agents represent a considerably safer and low toxicity alternative to synthetic fungicides. In the present review, these biological control strategies as alternative to the chemical fungicides are summarized here and new challenges regarding the development of shelf-stable formulated biocontrol products are also discussed.

## 1. Introduction

Citrus is one of the most produced fruit genus. Grown in more than 100 countries, this group is composed by several species, including oranges, tangerines, mandarins, grapefruits, lemons, and limes [1]. The impact of citrus agroindustry in the international economy is huge. Besides their value as commodities, they also provide employment in many segments involved in its production cycle: harvesting, handling, transportation, and storage. In 2017, the global orange production reached 47.6 million metric tons (tons) and is expected to expand 4.2 million in 2018/2019 due to favorable weather in Brazil and United States, two of the most important orange producers in the world [2]. 

Over 20 different kinds of postharvest diseases have been reported in citrus and they are the main cause of fruit spoilage, resulting in massive economic losses [3]. Moreover, fungal growth in fruit may lead to production of mycotoxins, including potential carcinogenic agents such as citrinin and patulin [4], as well as tremorgenic compounds, for example tryptoquivalines [5], therefore representing a threat to human and animal life.

Green mold, blue mold, and sour rot, caused by *Penicillium digitatum, P. italicum*, and *Geotrichum citri-aurantii,* respectively, are the main citrus postharvest diseases [6]. *P. digitatum*, alone, is responsible for approximately 90% of total postharvest losses [7]. The fruits are contaminated through skin postharvest damage during their picking, packaging, storage, and transportation [3].

The interaction between citrus fruit and these phytopathogens is not fully understood, but some factors are known to affect this interaction in order to increase the fungus pathogenicity. *P. digitatum* and *P. italicum* are known to secrete organic acids during infection, leading to an optimal pH for its cell wall-degrading enzymes, such as polygalacturonases (PG) [8,9]. Moreover, *P. digitatum* also produce catalase during infection, an antioxidant enzyme that decomposes hydrogen peroxide, the main defense mechanism in citrus [10].

In order to deal with these fungi, chemical fungicides have been the main focus of research over the past decades. *P. digitatum* and *P. italicum* can be efficiently controlled by imazalil, thiabendazole, or pyrimethanil, but these fungicides are not effective against sour rot. For the control of *G. citri-aurantii*, guazatine, and propiconazole can be applied, although they are not allowed in some producing countries such as Brazil [11]. However, widespread use of chemical fungicides has caused the proliferation of resistant strains of these phytophatogens, compromising the effectiveness of these treatments [12]. Furthermore, concerns about environmental contamination and risks associated to human health have been raised around the accumulation of their residues in food. 

Finding commercially viable, effective, alternative control methods has been a leading challenge for researchers, especially for controlling *G. citri-aurantii*, since there are fewer options available of acceptable chemical fungicides. Many alternatives have been proposed, including the application of antagonistic microorganisms and natural antimicrobial substances. Natural antimicrobial substances, especially plant extracts, are considered relatively safe, presenting low toxicity and high decomposability due to their natural origin, raising particular interest for use in these natural products [13].

Thus, the application of biocontrol agents has been an alternative for synthetic chemical fungicides. However, more research is necessary to understand their mechanism of action and effectiveness in different infection levels; this knowledge is crucial to implement their use as practical control agents. Therefore, the use of alternative postharvest biological control methods, both non-polluting and possessing low toxicity are reviewed here, highlighting advances presented in the literature in the recent years.

## 2. Alternative Control Methods

### 2.1. Microrganisms

Besides fungicides, other agricultural practices such as irradiation application (light emitting diode, gamma radiation, or UV radiation) [14,15,16,17], thermotherapy [18,19,20], biocontrol agents (BCA) [21,22,23], and salt solution [24,25] may be used to control postharvest diseases. In the past thirty years, there have been extensive research activities to explore and develop strategies based on microbial antagonists to biologically control postharvest pathogens [26,27,28,29,30]. This section focuses on the natural products and BCA described to be effective to control postharvest diseases.

BCA have been used in post-harvested fruits and its mechanism of action is poorly described in the literature for the majority of microorganisms (either bacteria or yeasts). However, it is supposed that more than one control mechanism could be acting simultaneously over the host-pathogen-antagonist and environment interactions [30]; others include: antibiosis [30,31], competition for nutrients or space [32], induction of resistance in citrus fruits [33,34], secretion of specific enzymes [35], stimulation of ROS in host tissues [36], mycoparasitism, and biofilm formation [37,38]. 

The use of yeasts as antagonists has been extensively studied, due to their high inhibitory capacity and the ability to colonize surfaces for a long period. The so-called “killer yeasts” yeast strains have the ‘killer’ phenotype (K+) and can produce the “killer proteins” that are potential antifungal agents; this feature is a biological advantage against others competing microbial [39,40,41]. Ferraz et al. reported that *Rhodotorula minuta, Candida azyma*, and *Aureobasidium pullulans* presented killer activity against the citrus pathogen *G. citri-aurantii*, deforming fungal hyphae and suppressing pathogen development [11]. *Saccharomyces cerevisiae* is another example of yeast that often presents killer activity [40,42,43,44]. 

Besides yeasts, bacteria are also promising BCA: *Bacillus* [45,46], *Lactobacillus* [47,48], and *Streptomyces sp.* [49,50] genus have been studied as BCA against citrus pathogens. Bacteria of *Bacillus* genus can act as antagonist through antibiotics or volatile organic compound (VOC) production that can induce the increase of plants resistance. Leelasuphakul et al. verified that strains of *Bacillus subtilis* found in soil were able to delay the spore germination of *Penicillium digitatum* by the action of water-soluble antibiotic secondary metabolites, proteins, enzymes, and VOC production [45]. As for *Lactobacillus*, metabolites such as 3-phenyllactic acid and allyl phenylacetate isolated from *L. plantarum* IMAU10014 had their antifungal activities against *Penicillium digitatum* observed *in vitro* by Wang et al. [47]. Finally, *Streptomyces sp.* had been tested *in vitro* and *in vivo* against *P. digitatum* and other pathogens [49,50]. Metabolites from *Streptomyces* RO3 cultures with molecular masses higher than 2000 Da showed fungicidal action and *in vivo* tests indicated that the green mold disease incidence decreased when treated with *Streptomyces* RO3 metabolites [49].

Among the alternatives reported in the literature, the induction of host resistance to pathogen by microorganisms with ‘killer’ activity has been pointed as a promising option for plant disease control, since they are active against a broad spectrum of pathogens and are safer than other alternatives [41,51]. For example, Parafati et al. related that the yeasts *Wickerhamomyces anomalus, Metschnikowia pulcherrima,* and *A. pullulans* increased the activities of peroxidase and superoxide dismutase in mandarins, reducing the incidence and severity of blue mold on these fruits [52]. Unfortunately, the activities and mechanisms of interaction of most ‘killers’ have not yet been well elucidated [11,41,44], being an open research field.

Another gap in the search for BCA against citrus pathogens is that the majority of the studies focuses only in *P. digitatum* and the other citrus pathogens such as *P. italicum* and *G. citri-aurantii* are less studied and also represent a problem for citriculture. As mentioned before, there are few fungicides active against sour rot and other approaches such as BCA could be explored to discover alternative methods to control this phytopathogen. However, few BCA are reported to control this disease. One possibility to solve this problem could be the evaluation of BCA already pointed as active against *P. digitatum* or other phytopathogens to control *G. citri-aurantii*.

Table 1 lists some known biocontrol agents and their mode of action against *P. digitatum*, *P. italicum*, and *G. citri-aurantii*.

### 2.2. Natural Products

Besides BCA, the use of chemicals isolated from natural sources may be an interesting approach to control *P. digitatum*; these include alkaloids [62], chitosan [26,63,64,65,66], carvacrol and thymol [67], citral [68], citronellal [69], and several other compounds isolated from essential oils (EOs) and plant extracts [22,70,71].

Olmedo et al. assessed the antifungal activity of six β-carboline alkaloids (harmine, harmol, norharmane, harmane, harmaline, and harmalol) against *P. digitatum* and *Botrytis cinerea*. They observed that harmol is more active than harmaline and harmalol, due to the differences in properties such as aromaticity, acidity, planarity, and polarity [62]. Table 2 shows some plant natural products that have been currently studied as control strategies against *P. digitatum, P. italicum*, and *G. citri-aurantii*; lists that are more extensive can be found in [22,71].

**Figure 1:**
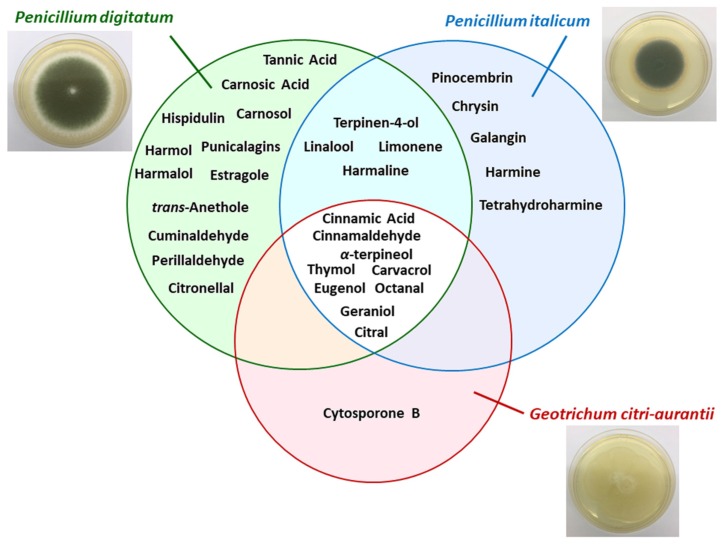
Venn diagram comparing the number of active natural products against the different postharvest citrus pathogens.

The natural products highlighted in Table 2 are summarized in Figure 1. The global Venn diagram (Figure 1) displays natural products activity distribution against *P. italicum*, *P. digitatum*, and *G. citri-aurantii.* The Venn diagram clearly indicates a significant number of natural products active against *P. digitatum* and *P. italicum*; however, few natural products have been studied to control *G. citri-aurantii.*

Garlic [88], neem [89], *Withania somnifera L.* and *Acacia seyal L.* [87], mustard, and radish [90] also have been reported as effective in controlling *P. digitatum*. Recently, Zhu et al. reported the antifungal activity of tannic acid on *P. digitatum*. *In vivo* tests showed significant decrease of disease signals of *P. digitatum* by inhibiting its mycelial growth and spore germination. Storage tests shown that tannins reduce the severity of green mold on citrus by 70%. The authors suggest that antifungal activity mechanism of tannic acid is related to the disruption of the cell walls and the plasmatic membrane, causing leakage of intracellular contents such as sugars [82].

Extracts from chili peppers and ginger were also proposed to control or inhibit postharvest diseases in citrus [91]. Singh et al. tested different concentrations of the plant extracts of the *Zingier officinale L.* (Ginger) and *Capsicum frutescence L.* (Chilly) against *P. digitatum*, *Aspergillus niger*, and *Fusarium* sp. isolated from naturally infected citrus. *P. digitatum*, specifically, had a reduction on colony development with inhibition zones of 51.5%, 69.2%, 74%, and 83.1% for *Zingier officinale L.* extract, and 56.4%, 64,1%, 76.6%, and 100% for *Capsicum frutescence L.* at concentration of 500, 1000, 2000, and 3000 ppm, respectively [91].

Besides the above-mentioned natural products used against *P. digitatum*, synergism between compounds have also been tested. Shi et al. studied the effects of chitosan and salicylic acid (SA), both isolated and mixed, on the control of green mold decay in grapefruit. The results showed that combination of chitosan with SA was effective to control green mold than either compound alone (significant efficacy of biocontrol agents against green mold decay induced by SA application in citrus fruits have previously been studied) [26]. Furthermore, significant reduction on lesion diameter and disease incidence was observed. Additionally, it was observed that treatment with chitosan/SA blends increased the content on ascorbic acid and total soluble solids in the fruits, providing a longer shelf life [26].

*P. italicum* causes the blue mold decay and represents one of the most problematic postharvest citrus infection, compromising fruit integrity during storage and transportation [92]. Currently, the blue mold is primarily controlled by the synthetic fungicide applications, such as thiabendazole and imazalil [44]. Regarding Imazalil-resistant biotypes *P. digitatum* is more common, whereas resistant *P. italicum* is rare [93]. 

Plant extracts studied as an alternative or complementary control agents to currently used fungicides may be attractive because of their potential antifungal activity, non-phytotoxicity and biodegradability [94,95,96,97]. Askarne et al. evaluated the antifungal activity of 50 species of plants collected in different regions of southern Morocco. *In vitro* antifungal activity showed that among them, *Anvillea radiata* and *Thymus leptobotrys* completely inhibited mycelial growth of *P. italicum* at concentrations of 10% m/v [98]. In addition, *Asteriscus graveolens*, *Bubonium odorum*, *Ighermia pinifolia*, *Inula viscosa*, *Halimium umbellatum*, *Hammada scoparia*, *Rubus ulmifolius*, *Sanguisorba minor*, and *Ceratonia siliqua* were also effective against *P. italicum* with inhibition of mycelial growth greater than 75%. The species on *in vitro* studies were also tested *in vivo* against the blue mold in citrus. The incidence of blue mold was significantly reduced to 5 and 25% when oranges were treated with aqueous extracts of *H. umbellatum* and *I. viscosa* (compared to 98% in the control), indicating the antifungal potential of these materials against *P. italicum*.

Kanan and Al-Najar reported the effective *in vitro* and *in vivo* antifungal activity of fenugreek (*Trigonella foenum-graecum L*.), harmal seeds (*Peganum harmala L.*), garlic cloves (*Allium sativum L.*), cinnamon bark (*Cinnamomum cassia L.*), sticky fleabane leaves (*Inula viscose L.*), nightshade leaves, and fruits (*Solanum nigrum L.*) against *P. italicum* isolates. Cinnamon, garlic, and sticky fleabane methanolic fractions resulted in complete inhibition of this pathogen [76].

The high antifungal activity against *P. italicum* of crude extracts cinnamon, as well as the corresponding methanolic, hexanic, and aqueous fractions was related to the high content of cinnamaldehyde, eugenol, cinnamic acid, flavonoids, alkaloids, tannins, anthraquinones, and phenolic compounds, some of them reported before as active antifungal agents. Among them, eugenol and cinnamaldehyde have been consistently reported to be the main antifungal components of cinnamon [79,99], representing potential, environmentally benign candidates for postharvest disease control. In addition, harmal extract was pointed also as highly effective extract against *P. italicum in vitro*. The activity of the harmal crude extracts may be related to the content of alkaloids such as harmine, harmaline, and tetrahydroharmine besides phenolic compounds that can alter the permeability of fungal cells [76]. The exact mechanism of action of phenols has not yet been determined; however, it is already known that they can inactivate essential enzymes and disrupt function of the genetic material [100].

Several studies have reported natural antifungal compounds against *P. italicum* produced by different sources. Essential oils, for instance, represent a low-toxic effects alternative and are reported to possess strong inhibitory effects on crop contaminated by *P. italicum* [74]. Among the major volatile constituents are limonene, β-linalool, α-terpineol, citral, and octanal, the latter reportedly exhibits antifungal activity against some postharvest pathogens such as *P. digitatum, P. italicum*, and *P. ulaiense* [101,102]. Regarding the mode of action, Tao et al. attributed its activity against mycelial growth of *P. italicum* and *P. digitatum* to the disruption of the cell membrane integrity and leakage of ions and other cell contents [74]. A similar mechanism of activity against postharvest citrus pathogens was attributed to citral, present in citrus EO that is able to alter the morphology of *P. italicum* hyphae [75].

Chinese propolis has been pointed as active against blue mold and the flavonoid pinocembrin (5,7-dihydroxyflavanone) was identified as one of its main active antifungal constituents. Peng et al. studied the inhibitory effect of this compound on *P. italicum* with particular attention to its response to the mycelial growth and energy metabolism by interfering in energy homeostasis and cell membrane damage of the pathogen. They observed that mycelial growth and spore germination were nearly completely inhibited with inhibitive percentage up to 93 and 97%, respectively, for pinocembrin concentrations of 400 mg/L [72].

**Figure 2:**
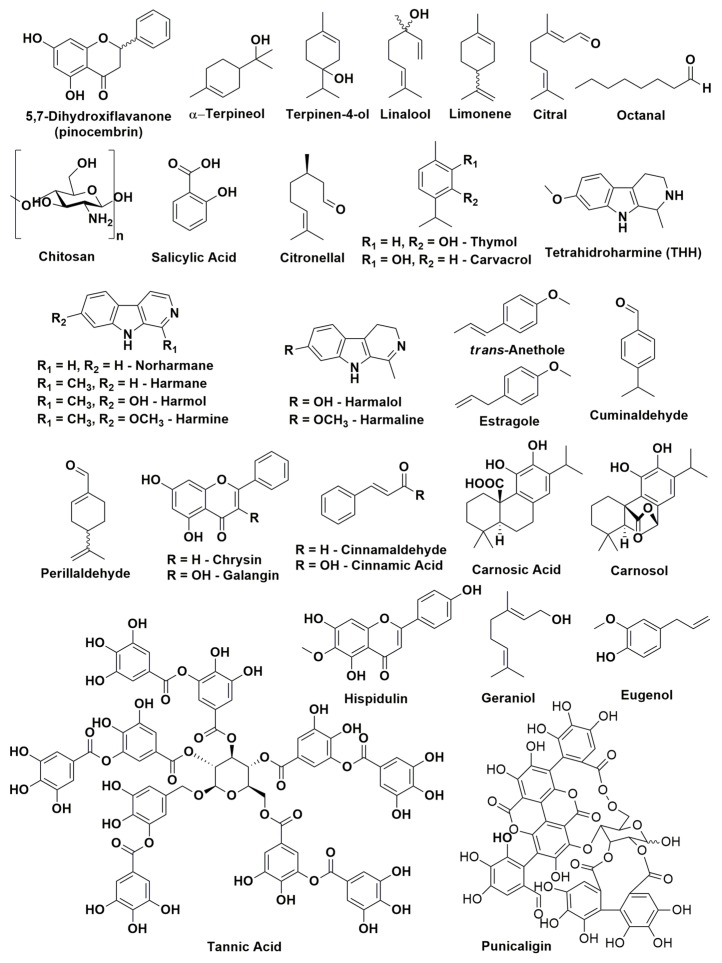
Chemical structures of some antifungal compounds active against *P. digitatum* and *P. italicum* found in essential oils and natural extracts.

Figure 2 shows the structures of the above mentioned antifungal compounds found in natural extracts and essential oils.

Sour rot of citrus, caused by *Geotrichum citri-aurantii*, represents another potentially devastating storage disease [103]. Pathogenicity of *Geotrichum citri-aurantii* on citrus fruit involves secretion of extracellular endo-polygalacturonases (PG) that aid in the rapid breakdown of infected tissues facilitating the disease [104]. The usual fungicides, with the partial exception of sodium o-phenylphenate (SOPP) and propiconazole [105], cannot actively controlled this disease. Yin et al. revealed the first time that cytosporone B—a compound isolated from the endophytic fungus *Cytospora sp.* and presenting a wide range of antitumor and antimicrobiotic activities—has a promising effect on the control of citrus decay caused by this pathogen, being comparable to that of fungicide prochloraz. Its mechanism of action is suggested to be related to the alteration of the morphology of pathogen cells, causing distortion of the mycelia and loss of membrane integrity [106].

Talibi et al. demonstrated that methanolic extracts *Cistus villosus*, *C. siliqua*, and *H. umbellatum* successfully reduced the disease incidence *in vitro* caused by *G. citri-aurantii* with no phytotoxic effects recorded on citrus; on the other hand, ethyl acetate extracts of *A. radiata, C. villosus*, and *C. siliqua* proved to be the best inhibitors of mycelial growth [107]. Other studies concerning the antifungal properties of organic extracts of plants include materials from *Cistus L.* species [108] and from extremophile plants from the Argentine Puna [109]. Incidence of sour rot was described to reduce significantly when fruits were treated with *Cistus populifolius* and *Cistus ladanifer* methanol extracts [108].

Zhou et al. also reported citral, octanal, and α-terpineol to have strong inhibition on *G. citri-aurantii*, being the former the most potent among them. They induced a decrease on the total lipid content of the cells, indicating the destruction of cellular membranes, disruption of cell membrane integrity, and leakage of cell components [110].

Liu et al. studied the antifungal activity of thyme (*Thymus sp.*) EO against postharvest sour rot on citrus fruit. It was shown that *G. citri-aurantii* cells treated with thyme EO showed morphology alteration (collapsed mycelia and arthroconidia structures); additionally, a marked enlargement of hypha wall thickness was observed [111]. Those effects can be assigned specially to the presence of thymol (a volatile terpenoid ubiquous in plants with strong, widespread antifungal activities).

Regnier et al. screened 59 commercially available EO, as well as their major components, to determine their effects on mycelial growth of *G. citri-aurantii* [6]. Lemong (*Cymbopogon citratus*) was found to be the most cost-effective option; it was also found that a blend of the lemongrass and spearmint (*Mentha spicata*) EO could be an alternative for effective multi-target protection against *G. citri-aurantii*, *P. digitatum*, and *P. italicum* [6].

Xu et al. found that cassia (*Cassia sp.*) EOs have the ability to control postharvest pathogens and diseases, but their poor solubility in water might prevent its effective use. In order to circumvent this problem, they presented an aqueous microemulsion formulation of cassia EO with ethanol as co-surfactant and Tween 20 as surfactant as antifungal agent against *G. citri-aurantii*. Both *in vitro* and *in vivo* assays showed that cassia EO had stronger activity when encapsulated in the microemulsion [112].

**Figure 3:**
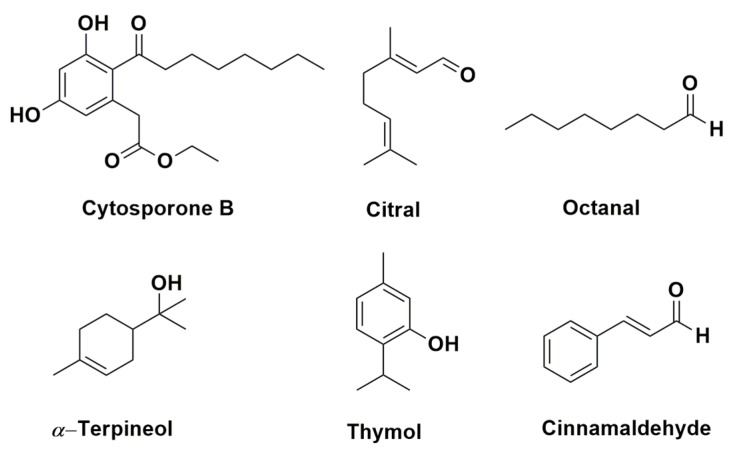
Chemical structures of compound found on plant extracts and essential oils with activity against *G. citri-aurantii*.

The structure of some compounds above listed with activity against *G. citri-aurantii* are shown in Figure 3.

Considering that most plant-derived compounds are usually much less toxic to humans and fauna in general, as well as generally environmentally-friend when compared to fungicides, they should be considered safer and may represent a promising alternative to the existing chemical pesticides in the control of fungal diseases. However, it should be noted that there is a lack of studies concerning non-chemical postharvest treatment against *G. citri-aurantii* and very little research has been carried out to investigate the use of natural products to control citrus sour rot; therefore, special attention towards this pathogen is needed. Despite the promising antifungal activity of organic extracts of plants, the active antifungal compounds are frequently not identified; the knowledge of the substances accountable for the antifungal activity is fundamental to formulate viable commercial products and develop advanced biofungicides formulations as alternatives to synthetic fungicides to control the citrus postharvest diseases.

### 2.3. Commercial Biofungicides

Biofungicide is the general name given to microorganisms and naturally occurring compounds that possess the ability to control plant diseases [113]. Although the mechanisms of biocontrol in postharvest diseases have not been fully explained in many cases, effective colonization of wounds and competition for nutrients appear to be significant factors for many antagonists. Many microorganisms have shown good potential as basis for commercial biocontrol products due to their efficacy against fungal pathogens in field conditions. Although much research is being conducted in this area, a limited number of biofungicides are available commercially [114,115,116].

Several commercial biological control formulations based on *Trichoderma harzianum, A. pullulans, Bacillus subtilis, Streptomyces griseoviridis*, and *Gliocladium virens* [113,117] have been reported for application against different plant fungal pathogens. Most studies have focused on application of individual biocontrol agents without evaluating their combination with other microorganisms or even with chemical components. Nevertheless, combination BCA that are compatible with each other could offer a new and effective approach improving plant diseases control [114,118].

The major drawback in the commercialization of bioproducts based on BCA is the advancement in the production of shelf-stable formulated products that maintain biocontrol activity similar to that of the fresh cells. Although biofungicides have good action background against host pathogens, there are limitations to their use and effectiveness in the field. Growing demand and interest in bioproducts have led to many marketable brands but the absence of field application reliability of biofungicides has been a significant obstacle in the adoption of these approaches. Despite the remarkable results obtained with biofungicides in the laboratory experiments, some failed to provide consistent disease control in field conditions. Thus, these factors are potential contributors to the low dissemination of these products on the market [113,115,116].

**Figure 4:**
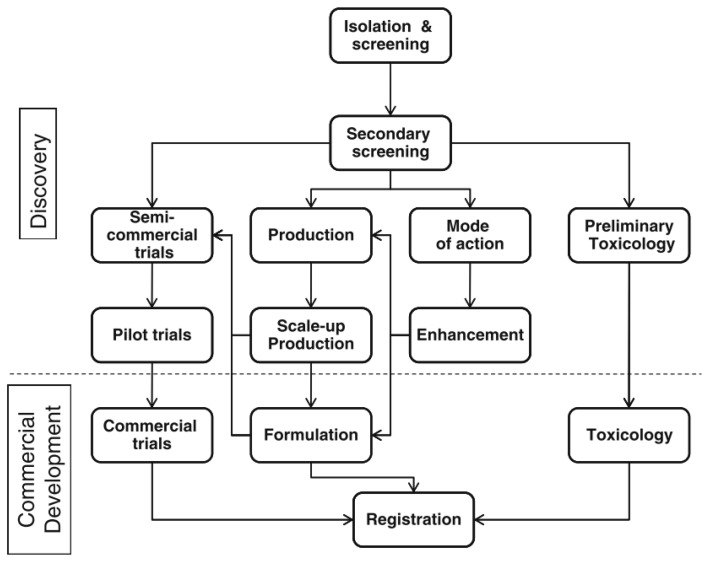
Outline flow of development of a postharvest biocontrol agent. Reprinted from [119]. Copyright 2012, Springer Nature.

Although a reasonable number of studies take into account the exploration of new bioactive compounds that may specifically act against citrus phytopathogens, few products come to commercialization and are widely marketed. In addition to the above problems, the workflow that starts with the discovery of the bioactive compound to the effective elaboration of the final product is still quite complex and is usually a long, iterative process that involves several steps. Figure 4 shows a usual workflow that could be involved in the development of a postharvest biofungicide.

According to Nunes, biocontrol agent development is comprised of two main steps: discovery and commercial development. Summarizing the required phases, the first one concerns the isolation and efficacy of the laboratory-based compounds to pilot tests. In this phase must be included evaluation of the action mechanism of the microorganisms, growth media required, improvements in biocontrol activity, and legal procedures involving the patent for the employment of the microorganism as a biopesticide. The commercial development of BCA involves steps including scale-up production, product formulation, biosafety of the microorganism, and registration [119]. The detailed workflow will not be discussed in detail but can be found in previous papers [116,119,120,121].

Indeed, the complex workflow is a weak point for the development of new products based on biomolecules. Moreover, the high cost of the production and the regulatory barriers to BCA registration in different countries do not encourage their dissemination and recurring issues that need to be overcome [119,120,121,122]. To date, four commercially biofungicides, based on microorganisms, were found for the control of postharvest citrus fruit. The yeast *Metschnikowia fructicola*, for instance, was reported as an efficient biological control agent of postharvest diseases of fruits and vegetables, and it is the bases of the commercial formulated product “Shemer” [122]. Its effect has been reported as a good control of decay in oranges being equivalent to oranges treated with the chemical fungicide, imazalil [123]. Nevertheless, just a few antagonists have achieved the commercial development stage as commercial products. Some of these biofungicides are represented in Table 3.

Aspire™, based on *Candida oleophila* [117], has been commercialized for some years but did not prevail due to low and inconsistent efficacy under commercial conditions, difficulties in market penetration, and perception of the customers and industry [113]. Other products, such as Shemer™ (based on the yeast *M. fructicola*), have been more successful [27], and are being used for both pre- and postharvest application on various fruits and vegetables, including citrus fruit, grapes, peaches, peppers, strawberries, and sweet potatoes.

Despite limited numbers of specific biofungicides, the demand for these products in agriculture as alternatives to synthetic pesticides has increased over the last few years. The adoption and widespread use of biofungicide will make it possible to produce food that are exempt from or have low values of chemical residues. This will contribute to the consumption of more natural, healthy, and safe foods with respect to fungicide usage [116,127]. Thus, both regulatory barriers and workflow-related procedures must be improved in order to overcome the challenges in the biofungicide market.

## 3. Conclusions

The information presented in this review reports the potential of alternative methods for the control of postharvest citrus diseases. Despite all the drawbacks regarding the development of new products, a considerable number of studies have been conducted concerning biocontrol strategies of citrus postharvest phytopathogens. Several studies have reported antifungal compounds, mostly against *P. digitatum*, which is responsible for the most important disease found in citrus fruits. Another interesting approach, with good results against green mold, is the application of the biocontrol agent in a mixture with low doses of chemical fungicides during citrus fruit processing. Nevertheless, few studies treat individually the particularities of each fungus and, therefore, many strategies of biocontrol still need to be studied taking into account the different metabolic and enzymatic fungi profile. In this context, there is a lack of studies on postharvest non-chemical treatment against *G. citri-aurantii*, which causes a decay not controlled by the conventional treatments, and therefore special attention toward this pathogen is necessary. It is very clear that in recent years the interest in biocontrol strategies that minimize the use of chemical pesticides is a worldwide trend, which has driven research in this field. Compounds from biological sources are usually much less toxic to humans as well as environmentally-friendly when compared to synthetic fungicides; for this reason they represent a promising alternative to the existing chemical pesticides in the treatment of fungal diseases. However, as discussed here, several challenges related to the workflow procedures and development of biofungicides still need to be overcome so that new technology can be employed during citrus fruit processing in order to lead to a commercially viable strategy that meets the needs of the producers.

## Figures and Tables

**Table 1:** Biocontrol agents (BCA) used against *P. digitatum*, *P. italicum*, and *G. citri-aurantii*.

Antagonist	Agent	Mechanism	Target Pathogen	References
Yeast	*Wickerhamomyces anomalus* (or *Pichia anomala*)	Antibiosis, competition for nutrients, fruit resistance induction and ‘killer’ activity	*P. digitatum P. italicum*	[39,40,41,52]
	*Saccharomyces cerevisiae*	Competition for nutrients or space and ‘killer’ activity	*P. digitatum P. italicum*	[40,41,43,44]
	*Candida oleophila*	Resistance induction. Increase phenylalanine ammonia lyase activity and accumulation of the phytoalexins such as scoparone, scopoletin, and umbelliferone	*P. digitatum P. italicum*	[33,53]
	*Saccharomycopsis crataegensis* + sodium bicarbonate	Not specified	*P. digitatum*	[54]
	*Kluyveromyces marxianus* + sodium bicarbonate	Competition for nutrient and space. The salt stimulates *K. marxianus* growth and it inhibits fungal spore germination	*P. digitatum*	[55]
	*Rhodosporidium paludigenum*	Fruit resistance induction. Increase in ethylene production and expression of defensive genes	*P. digitatum*	[56]
	*Pichia membranifaciens*	Competition for nutrients or space	*P. digitatum*	[57]
	*Metschnikowia pulcherrima,* and *Aureobasidium pullulans*	Competition for nutrients and fruit resistance induction by influencing peroxidase and superoxide dismutase activities	*P. digitatum P. italicum*	[52]
	*Candida stellimalicola*	‘Killer’ activity, production of chitinase, and inhibition of conidial germination	*P. italicum*	[44]
	*Cryptococcus laurentii* associated with cinnamic acid	Different influence of cinnamic acid on the antagonistic yeast and the pathogen, leading to synergistic effect	*P. italicum*	[58]
	*Metschnikowia citriensis*	Biofilm formation, adhesion to mycelia, and iron depletion	*P. digitatum P. italicum*	[53]
	*Pseudozyma antarctica*	Direct parasitism	*P. digitatum P. italicum*	[53]
	*Rhodotorula minuta*, *Candida azyma*, and *Aureobasidium pullulans*	‘Killer’ activity and hydrolytic enzyme production	*G. citri-aurantii*	[11]
	*Debaryomyces hansenii*	Competition for space and nutrients	*P. digitatum P. italicum*	[32,59,60]
	*Kazachstania exígua* and *Pichia fermentans*	‘Killer’ activity	*P. digitatum P. italicum*	[41]
	*Bacillus subtilis*	Water soluble antibiotics, proteins, enzymes, and VOC production	*P. digitatum*	[43,45]
Bacteria	*Bacillus amyloliquefaciens*	Great amounts of antibiotics produced *in vitro*, however, still not effective for green mold control *in vivo*	*P. digitatum*	[46]
	*Lactobacillus plantarum*	Metabolites 3-phenyllactic acid and benzeneacetic acid, 2-propenyl ester with antifungal activity	*P. digitatum*	[47,48]
	*Streptomyces sp.*	Metabolites with higher mass than 2000 and fungicidal effect	*P. digitatum G. citri-aurantii*	[49,50]
	*Streptomyces violascens*	Extracellular antifungal compounds that inhibits fungal spore germination and antibiosis	*G. citri-aurantii*	[61]

**Table 2:** Natural products extracted in plants as control strategies against *P. digitatum, P. italicum*, and *G. citri-aurantii.*

Plant/Fruit	Pathogen (s)	Extract/Method	Natural Products	Details	References
Chinese propolis	*P. italicum*	1) Ethyl acetate (3 times); 2) chloroform; 3) ethanol and water; 4) methanol	Pinocembrin	Pinocembrin acts against *P. italicum* through inhibition on respiration and interference of energy homeostasis	[72]
*Citrus aurantium*	*P. digitatum P. italicum*	Hydrodistillation (peels, leaves, and flowers)	α-terpineol, terpinen-4-ol, linalool, and limonene	Essential oils (EOs) of flowers and leaves reduced the growth of pathogen, while EO of peels was inactive	[73]
*Citrus eticulate Blanco*	*P. digitatum*	-	Citral	Antifungal activity of citral was tested *in vitro* and *in vivo* and combined with the wax showed potential for control applications	[68]
Citrus fruits	*P. italicum P. digitatum*	Commercial product	Octanal	Octanal inhibits the fungal mycelial growth	[74]
Citrus fruits	*P. italicum*	Commercial product	Citral	Citral inhibits the mycelial growth of *P. italicum* causing disruption of cell membrane integrity	[75]
*Citrus paradise* Macf. (Grapefruit fruit)	*P. digitatum*	-	Chitosan and salicylic acid	Chitosan combined with salicylic acid had better treatment of green mold than these isolated compounds, without compromising the quality of fruit.	[26]
*Citrus sinensis Osbeck*	*P. digitatum*	Commercial product	Citronellal	Citronellal was able to inhibit spores germination and mycelial growth. Just as citral, the compound combined with wax reduced the incidence rate	[69]
*Laminaceae spp.*	*P. digitatum P. italicum*	-	Carvacrol and thymol	The mechanisms that have been proposed for these compounds are: 1) morphological deformation and deterioration of the conidia and hyphae; 2) hydroxyl group and systems with delocalized electrons has important role for antimicrobial effect	[69]
*Peganum harmala L.* (harmal seeds)	*P. italicum*	Ethanol	Harmine, harmaline, and tetrahydroharmine (THH)	Harmal extracts showed strong antifungal activity against *P. italicum* and its activity is related to alkaloids harmine, harmaline e THH	[76]
*Peganum harmala L.*	*P. digitatum*	Commercial product	Harmol, harmaline, harmalol, harmane, and norharmane	It was tested the antifungal activity of *β*-carbolines against *P. digitatum* and *Botrytis cinerea*. Harmol showed highest antifungal activity after 24 h.	[63]
*Pimpinella anisum* and *Carum carvi*	*P. digitatum*	Hydrodistillation (seeds)	trans-anethole, estragole (anise oil), cuminaldehyde, and perillaldehyde (black caraway)	EO were able *in vitro* of reduce the germination, the mycelial growth of pathogen and the incidence of disease symptoms	[77]
*Populus* × *euramericana cv. ‘Neva’* (poplar buds)	*P. italicum*	Dichloromethane	Flavonoids of pinocembrin, chrysin, and galangin	Antifungal compounds from poplar buds active fraction, identified by HPLC–MS, had antifungal effect in the fungal hyphae analyzed by scanning electron microscopy and transmission electron microscopy images	[78]
*Punica Granatum*	*P. digitatum*	Ethanol/water (4:1)	Phenolic compounds with a prevalence of punicalagins	Pomegranate peel extract has a broad range of antifungal activity	[13]
*Ramulus cinnamomi*	*P. digitatum P. italicum G. citri-aurantii*	Ethyl acetate and n-buthanol	Cinnamic acid and cinnamaldehyde	Through ^1^H-NMR-based metabolomics it was identified the extracts related to antifungal activity of *Ramulus cinnamomi* after 4, 8, and 12 h. The antifungal mechanism of cinnamaldehyde it was also analyzed by ^1^H-NMR	[79]
*Rosmarinus officinalis L.*	*P. digitatum*	Hydrodistillation (for EO) and methanol	Flavonoids, polyphenols, and essential oils	EO act in the fungal cells by disrupting the membrane permeability and the osmotic balance	[80]
*Salvia fruticosa Mill.*	*P. digitatum*	Ethyl acetate	Carnosic acid, carnosol, and hispidulin	Compounds that have antifungal properties, according to its compositions, structures/activity, and literature	[81]
*Sapium baccatum*	*P. digitatum*	Commercial product	Tannic acid	*In vitro* antifungal activity to *P. digitatum* was verified between 400 and 1000 µg mL^−1^ of tannic acid inoculated in Ponkan fruit was sufficient to inhibit the mycelial growth of 45% to 100%	[82]
*Solanum nigrum*	*P. digitatum*	Aqueous extract (leaves)	Alkaloids, flavonoids, saponins, steroids, glycosides, terpenoids, and tannins	Bioactive compounds that has pharmacological prospects for development of drugs	[83]
*Thymus* species (*T. leptobotris, T. riatarum, T. broussonnetii* subsp. *hannonis*, and *T. satureioides* subsp. *pseudomastichina*)	*P. digitatum P. italicum G. citri-aurantii*	Hydrodistillation	Thymol, carvacrol, geraniol, eugenol, octanal, and citral	EO of four *Thymus* species showed antifungal activity. Through GC–MS, MIC, and previous studies determined the principal active compounds	[84]
*Thymus leptobotris*	*P. digitatum P. italicum G. citri-aurantii*	Methanol, chloroform	Thymol and carvacrol	The antifungal screening from EO obtained from 21 plants showed that the EO from *Thymus leptobotris* had the highest fungistatic effect. The active compounds were identified in previous studies.	[85]
*Thymus vulgaris* L.	*P. italicum P. digitatum*	-	Thymol	EO of thyme inhibited the mycelium growth (MIC 0.13 µL mL^−1^) and spore germination (MIC 0.50 µL mL^−1^) *in vitro* and *in vivo*	[86]
*Withania somnifera* + *Acacia seyal*	*P. digitatum*	Methanol/acetone/water—7:7:1, *v/v* (dried plant powder—1:20 *w*/*v*)	Insoluble and soluble phenolic compounds	Application of plants extract (*W. somnifera* and *A. seyal*) in the sick host, induced plant resistance through change of phenolic concentration (phenylpropanoid pathway)	[87]

**Table 3:** Commercial biofungicides, based on microorganisms, for the control of postharvest citrus fruit.

Microorganism	Product	Targeted Pathogens	References
*Candida oleophila*	Aspire	*Botrytis*, *Penicillium*	[117]
*Metschnikowia fructicola*	Shemer	*Botrytis*, *Penicillium*, *Rhizopus*, *Aspergillus*	[124]
*Pantoea agglomerans*	Pantovital	*Penicillium*, *Botrytis*, *Monilinia*	[125]
*Pseudomonas syringae*	Biosave	*Penicillium*, *Botrytis*, *Mucor*	[126]
